# Electrospun Antimicrobial Polylactic Acid/Tea Polyphenol Nanofibers for Food-Packaging Applications

**DOI:** 10.3390/polym10050561

**Published:** 2018-05-22

**Authors:** Yaowen Liu, Xue Liang, Shuyao Wang, Wen Qin, Qing Zhang

**Affiliations:** 1 College of Food Science, Sichuan Agricultural University, Ya’an 625014, China; xue6liang17@163.com (X.L.); shuyaow@126.com (S.W.); qinwen@sicau.edu.cn (W.Q.); zhangqing@sicau.edu.cn (Q.Z.); 2 School of Materials Science and Engineering, Southwest Jiaotong University, Chengdu 610031, China

**Keywords:** polylactic acid, tea polyphenols, electrospinning, antimicrobial packaging

## Abstract

The development of new bioactive food-packaging materials that extend the shelf life of food is an important objective. Herein, we report the fabrication of four polylactic acid/tea polyphenol (PLA/TP) composite nanofibers, with PLA/TP ratios of 5:1, 4:1, 3:1, and 2:1, by electrospinning. The morphological quality of each sample was examined by scanning electron microscopy (SEM), and samples with higher TP content were found to be deeper in color. The samples were then examined by Fourier transform infrared (FTIR) spectroscopy to confirm the presence of TP. Examination of the mechanical properties of these fibers revealed that the presence of TP decreased both tensile strength and elongation at break; however, this decrease was only slight for the PLA/TP-3:1 composite fiber. The addition of TP influenced the hydrophilic–hydrophobic property and release behavior of the composite fibers, which significantly improved the antioxidant behavior of these samples, with 1,1-diphenyl-2-picrylhydrazyl (DPPH) radical-scavenging capacities of up to 95.07% ± 10.55% observed. Finally, antimicrobial activities against *Escherichia coli* and *Staphylococcus aureus* of up to 92.26% ± 5.93% and 94.58% ± 6.53%, respectively, were observed for the PLA/TP-3:1 composite fiber. The present study demonstrated that PLA/TP composite nanofibers can potentially be used for food-packaging applications that extend food shelf life.

## 1. Introduction

As standards of living have improved, many researchers have begun to explore the use of natural bioactive materials in food-packaging applications, such as polysaccharides (chitosan, alginates, celluloses, starch, etc.) and natural hydrocolloids (whey-protein isolate, whey-protein concentrate, etc.) [1,2]. As a result, materials that are biocompatible, biodegradable, green, and safe will lead future trends in the food-packaging industry. Bioactive materials are presently under the spotlight, as they can preserve the quality of foods and extend their shelf life. The most researched materials are those that are natural or synthetic and biodegradable, such as chitosan, polylactic acid (PLA), and starch. In addition to having low environmental impact and good biocompatibility, these materials are readily available and are therefore potentially widely applicable to food packaging [3,4].

Because of advantages that include easy isolation and processing and low environmental impact following degradation, PLA has already been widely used in biomedical [5] and food-packaging applications [6,7]. However, PLA products have poor mechanical and antibacterial properties. To tackle this issue, researchers have attempted to modify PLA in order to make it suitable for food-packaging purposes [8]. Salmieri et al. also employed the solvent-casting technique to prepare antimicrobial nanocomposites containing oregano essential oil. The resulting fibers were then converted into packaging that was applied on mixed vegetables for about 14 days; these fibers demonstrated a strong capacity to inhibit *Listeria monocytogenes* [9]. In a study of fibers fabricated from a ternary PLA/polyhydroxybutyrate/D-limonene composite, Arrieta et al. found that polyhydroxybutyrate (PHB) contributes to improved oxygen-barrier capacity and surface water resistance, while D-limonene improves elongation at break. They also examined the disintegrability of the ternary composite fibers under composting conditions, which revealed that PHB delays the process, while D-limonene accelerates it. They concluded that these modifications resulted in better overall PLA performance, with promising applications for food packaging [10].

However, PLA has limited potential in food-packaging applications, as it is not bioactive and has poor antimicrobial activity. Tea polyphenols (TPs) are a class of phenolic compound found in teas. They are antimicrobial and act by preventing microorganism attachment and by directly damaging cell structures. They are powerful inhibitors of the growth and proliferation of many microorganisms, in particular, gram-negative bacteria, gram-positive bacteria, and fungi [11,12]. In addition, TPs are good antioxidants that effectively slow down the oxidization of some foods, thereby extending their shelf life [13]. Discovering that green tea polyphenols have good antioxidant capacities but poor interfacial activities, Von et al. used β-lactoglobulin as an emulsifier to form nanocomplexes containing green tea polyphenols and demonstrated that the addition of green tea polyphenols contributed to the improved oxidative stability of fish-liver oil [14]. Zhang et al. investigated the properties of edible coatings developed by incorporating different concentrations of TPs into sodium alginate and demonstrated that fresh winter jujube (*Ziziphus jujube*) treated with these coatings can be preserved at ambient temperature for about 8 d; the fruit treated in this manner maintained its ascorbic acid and total phenol contents, as well as its antioxidant enzyme activity. They concluded that the composite coating has the potential to maintain the quality of fresh jujube at ambient temperature [15]. In a study of the antimicrobial activities of TPs and chitosan using the agar-dilution method, He et al. demonstrated that TPs effectively inhibit gram-negative bacteria. The authors proceeded to prepare ternary nisin/TP/chitosan composite fibers for preserving chilled mutton and found that the ternary composite fiber effectively extended the shelf life of chilled mutton to 6–18 days by efficiently inhibiting the growth of spoilage microorganisms and pathogens [16]. All of the above-mentioned studies demonstrated that TPs have outstanding antioxidant and antimicrobial activities and can serve as effective preservatives in food-packaging applications.

Electrospinning is currently a popular and easily accessible method for processing composite materials [17,18,19,20,21,22]. This method does not require high temperatures; consequently, active substances do not lose their activities owing to thermal sensitivities and other factors [23,24,25,26]. This advantage facilitates the incorporation of bioactive materials into polymers in order to produce nanometer-scale composite fiber packaging materials. In the present study, we blended PLA and TPs in different ratios and processed the blends into PLA/TP composite nanofibers by electrospinning. We examined the characteristics of the electrospinning preparations and then characterized the resulting PLA/TP composite nanofibers by scanning electron microscopy (SEM) and Fourier transform infrared (FTIR) spectroscopy. Mechanical and optical properties and DPPH radical-scavenging and antimicrobial capacities were also examined in order to systematically determine the effect of different TP contents on the performance of the PLA/TP composite nanofibers.

## 2. Materials and Methods

### 2.1. Materials

PLA slices (*M*_w_ = 7.6 kDa, *M*_w_/*M_n_* = 1.32) were procured from Shenzhen Esun Industrial Co., Ltd. (Shenzhen, China), Amber TP powder (98% purity) was procured from the College of Food Science, Sichuan Agricultural University (Yaan, China). Dichloromethane (DCM) and *N*,*N*-dimethylformamide (DMF), both of analytic purity, were procured from Sinopharm Chemical Reagent Co., Ltd. (Shanghai, China)

### 2.2. PLA and TP Blending for Electrospinning

In order to study the effect of the PLA/TP ratio on the morphology of the PLA/TP composite nanofibers, 5:1, 4:1, 3:1, and 2:1 (*w*/*w*) PLA/TP electrospinning solutions were prepared by dissolving the required masses of PLA and TA in 7:3 (*v*/*v*) DCM/DMF with even stirring (magnetic stirrer) at ambient temperature, such that the solute mass fraction was 10%.

### 2.3. Electrospinning

The solutions prepared as described above were electrospun as follows. The blended solution was loaded into a syringe fitted with a syringe needle with a 0.7 mm inner diameter that had been ground flat for use as a capillary tube for controlling ejection flow. The height of the syringe tip was adjusted such that it was aligned with the center of the aluminum-foil collector. The syringe was mounted on a syringe pump and connected to the positive pole of a high-voltage direct-current power supply. The aluminum-foil collector was connected to the negative pole of the power supply and grounded. The voltage was set to 20 kV, the distance was set to 15 cm, and the electrospinning speed was set to 20 mL/h. The collected fibers were vacuum dried for 24 h to remove solvent residue [27].

### 2.4. Characterization of Composite Fibers

The color features of the electrospun fibers were observed using an optical microscope (Nikon Eclipse TS100, Tokyo, Japan). The morphologies of the electrospun fibers were investigated by SEM (FEI Quanta 200, Eindhoven, The Netherlands) equipped with a field-emission gun (20 kV) and a Robinson detector after 2 min of gold coating to minimize the charging effect. ImageJ software (V 1.8.0) was then used to select 50 images from each of the desired samples, from which the fiber diameters were measured. Viscosity measurements were carried out using a rotary viscometer (NDJ279, Mechanical and Electrical Factory of Tongji University, Shanghai, China); they were performed at 25 °C. All the polymer solutions were used immediately after preparation. The interaction between PLA and TP was investigated using FITR spectroscopy. All the samples were recorded on a FTIR NICOLET iS10 spectrophotometer (FTIR NICOLET iS10, Waltham, MA, USA). The FTIR analysis was performed using the powder state of TP, which was mixed with KBr powder and palletized. A transmittance mode was used to obtain the FTIR absorption spectrum. For nanofibers, Attenuated Total Refraction (ATR) was used for FTIR measurement. The FTIR spectra analysis was performed in the mean infrared region with a wavenumber range of 4000–650 cm^−1^ by averaging 32 scans and using a spectral resolution of 4 cm^−1^. The mechanical properties of blends were obtained at 25 °C and 60% Relative Humidity (RH). The tensile strength (σb) and elongation at break (εb) of the fibers were measured on the electron tensile tester CMT-6104 (Shenzhen Sans Test Machine Co., Ltd., Shenzhen, China) to describe the mechanical behavior of the fibers. The fiber strips were cut into 60 × 10 mm strips, and the gauge length (i.e., the distance between the two clamps) was set at 40 mm, with a tensile rate (i.e., the rate of extending travel of the clamp) of 250 mm/min, a return rate (i.e., the rate of return travel of the clamp) of 200 mm/min, and a breaking load of 200 N. The contact angles of the PLA and PLA/TP fibers were measured by a video-based optical contact-angle meter (Data Physics OCA 15EC, Stuttgart, Germany). Briefly, about 6 μL of deionized water was dropped onto the fiber surface for the tests at a contact time of 5 s. The contact angles on the left and right sides of the drop were measured in software, and the average of 10 angles was reported for each sample. The release of TPs from the films was carried out by determining the active compounds from the polymer into the various food simulants: 95% ethanol as a simulant for fats, oil, and fatty foods [28] was used at 25 °C, and 50% ethanol as a simulant for oil in water emulsions and alcoholic beverages [29] was used at 4 °C. The food simulant (0.1 mL) was collected, and its TP content was determined at predetermined times (0, 10, 20, 30, 40, 50, and 60 h), in accordance with Liu et al. [30].

### 2.5. Antioxidant Activity

The antioxidant activity test, a DPPH method, was carried out for the PLA nanofibers and PLA/TP nanofibers. The radical-scavenging activities (RSAs) of the PLA nanofibers and PLA/TP nanofibers were evaluated using the DPPH assay [31]. Different samples (equivalent to 0.2 g nanofibers) were taken in test tubes. The total volume was adjusted to 8.5 mL by the addition of methanol; 5.0 mL of a 0.1 mM methanolic solution of DPPH was added to these tubes and was mixed well with a vortex mixer. The tubes were kept at room temperature for 20 min. The blank was prepared as above without the extract, and methanol was used for the baseline correction. Changes in the absorbance of the extract samples were measured at 517 nm via UV–VIS NIR (Ultraviolet Visible light Near Infrared) spectroscopy (Varian Cary 5000, Palo Alto, CA, USA). RSA was expressed as the inhibition percentage and was calculated using the following formula:

(1) $$\mathrm{RSA}\left(\%\right)=\frac{\mathrm{Absorbance}\;\mathrm{of}\;\mathrm{blank}-\mathrm{absorbance}\;\mathrm{of}\;\mathrm{samples}}{\mathrm{Absorbance}\;\mathrm{of}\;\mathrm{blank}}\times 100.$$

### 2.6. Antibacterial Testing

For the antibacterial tests, *Staphylococcus aureus* and *Escherichia coli*, representing gram-positive and -negative bacteria, respectively, were selected. The fiber samples from the experimental group and the control group were cut into pieces and placed into Erlenmeyer flasks, to which a growth medium and the bacterial strains were added. After 24 h of incubation, the bacterial colonies were counted. Next, the experimental group and the control group samples were shake-cultured for 24 h, then diluted and spread on a prepared solid culture medium, and cultured at a constant temperature for 24–48 h. Finally, the bacterial colonies were counted again and the value was recorded as the after-culture bacteria count. The antibacterial rate was calculated and averaged [23].

### 2.7. Statistical Analysis

One-way analysis of variance (ANOVA) was performed using Statgraphics Plus for Windows 5.1 (Manugistics Corp., Rockville, MD, USA). Fisher’s least significant difference (LSD) was used at the 95% confidence level.

## 3. Results and Discussion

### 3.1. Colors of the Electrospinning Solutions and Fiber Morphologies

**Figure 1:**
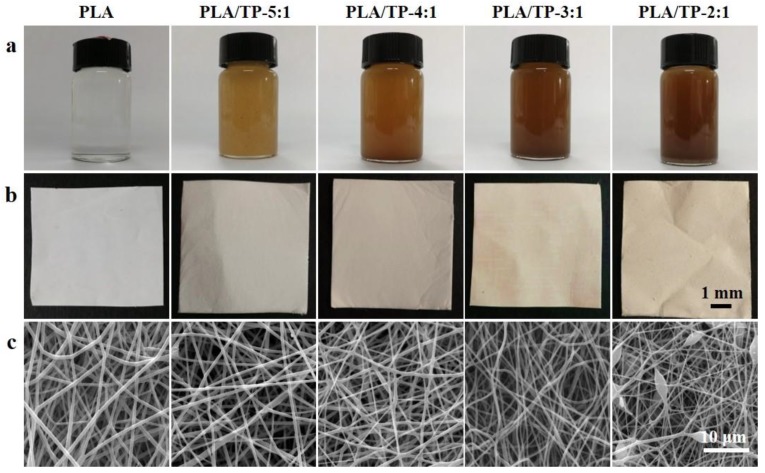
(**a**) Digital image of the polyactic acid (PLA) and PLA/tea polyphenol (TP) electrospinning solutions; (**b**) the appearance of the blended PLA and PLA/TP electrospun fibers; (**c**) scanning electron microscopy (SEM) images of the PLA and PLA/TP composite fibers.

As shown in Figure 1a, the pure PLA solution was colorless and transparent, while the color of the PLA/TP blend deepened with increasing TP content, as the pure TP powder was amber in color. After standing for 24 h, the quality of each blended solution was examined. No stratification was observed, which indicated that the PLA and TP were evenly suspended and dispersed in solutions prepared using all four ratios of components (5:1, 4:1, 3:1, and 2:1).

As the ratio of TP in the PLA fiber increased, the color of the blended electrospun fibers changed from gray to amber (Figure 1b). Figure 1c displays SEM images of the PLA/TP electrospun fibers. The images reveal that the electrospun fibers from all PLA/TP blends had smooth surfaces and relatively uniform fiber diameter distributions, with the exception of those produced with the 2:1 blend, which displayed bead defects. Perhaps the highly hydrophilic TP surface, which usually results in precipitation and aggregation of a polymer solution, was responsible for this observation. When the TP content was higher than that of the 2:1 blend, powder rather than fiber was collected on the aluminum foil (data not shown). This finding is consistent with the viscosities of the blended solutions reported below.

### 3.2. Viscosities and Conductivities of the Electrospinning Solutions and Fiber Diameters

**Figure 2:**
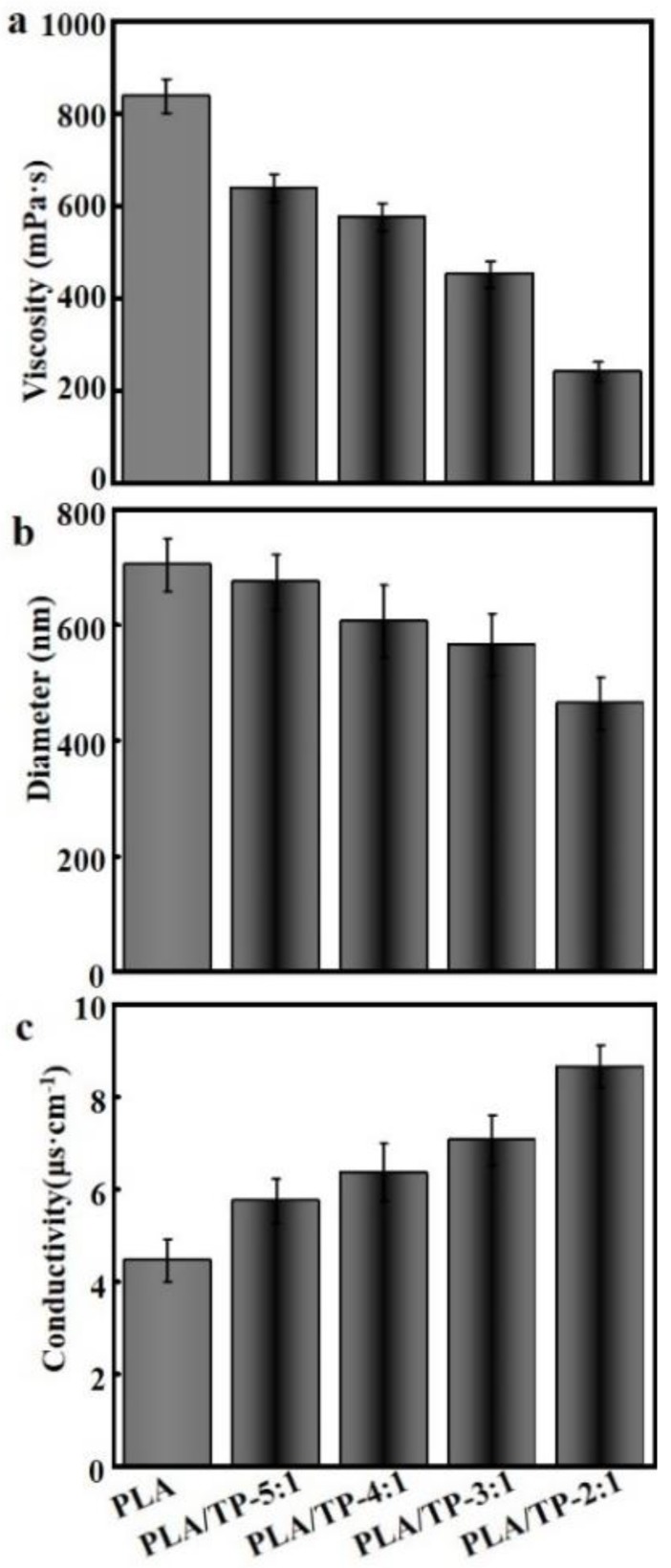
(**a**) Viscosities of different polylactic acid (PLA) electrospinning solutions; (**b**) diameter of different fibers determined from scanning electron microscopy (SEM) images using ImageJ software V1.8.0; (**c**) conductivities of the PLA electrospinning solutions containing different amounts of tea polyphenols (TPs).

The viscosities of all solutions were measured; Figure 2a reveals that the viscosity of the blend decreased with an increasing TP content. The viscosities of pure PLA and the 2:1 PLA/TP mixture were determined to be 835.7 ± 37.6 and 241.3 ± 14.6 mPa·s, respectively, and were clearly significantly different. Pelissari et al. demonstrated that the viscosity of a solution is associated with the interactions between the component molecules and depends on the concentrations and natures of the solutes and the reagents used, as well as on the pH [32]. Pelissari et al. also showed that the presence of TPs interferes with the inherent hydrogen bonding between molecules in a PLA solution, thereby weakening the bonds between its constituent components and resulting in a lower solution viscosity [32].

Figure 2b displays the diameters of different fibers, which reveals that the average fiber diameter decreased with increasing TP content, with the average value decreasing more sharply in moving from the pure PLA fiber (753 ± 84 nm) to the 2:1 fiber (493 ± 46 nm); all fiber diameters were homogeneously distributed. Fiber diameter was determined by multiple factors, including the solvent, polymer, polymer concentration, electrical conductivity of the solution, voltage, collection distance, and solution delivery rate. Generally, a decrease in the viscosity of an electrospinning solution leads to an increase in conductivity (Figure 2c), a decrease in surface tension, and a decrease in fiber diameter [33]. The outcome of the present experiment was consistent with the above-mentioned general rule. For instance, the viscosity decreased from 657.5 ± 28.4 mPa·s in the 5:1 solution to 241.3 ± 14.6 mPa·s in the 2:1 solution (Figure 2a), which was a result of the TPs possessing more polar groups than PLA. As the TP content in the spinning solution increased, the conductivity of the solution also increased (from 4.24 ± 84 nm), which, in turn, led to higher electrostatic repulsion between ejection flows and the formation of thinner ejection flows; ultimately smaller-diameter fibers were collected on the aluminum foil. On the other hand, increases in conductivity and decreases in surface tension may very possibly result in some of the ejection flows being too thin and over-stretched, which explains why bead defects and uneven fiber diameters were found in the 2:1 sample.

### 3.3. FTIR Spectroscopy

**Figure 3:**
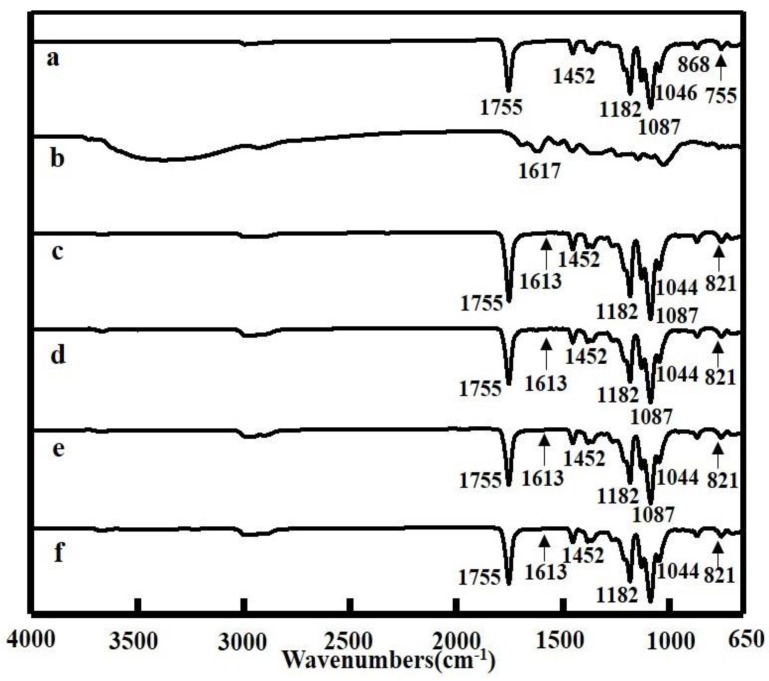
Fourier transform infrared (FTIR) spectra of polylactic acid (PLA) and PLA with different tea polyphenol (TP) contents: (**a**) PLA fiber; (**b**) TP powder; (**c**) PLA with TP (5:1); (**d**) PLA with TP (4:1); (**e**) PLA with TP (3:1); (**f**) PLA with TP (2:1).

Interactions between PLA and TP were investigated by FTIR spectroscopy. Figure 3 displays characteristic FTIR spectra of PLA, TP, and PLA/TP fibers. The infrared spectrum of PLA is characterized by the following absorption bands: 755 and 869 cm^−1^ due to C–H-bond stretches; 1045 and 1087 cm^−1^ due to C–O-bond deformation vibrations; 1182 cm^−1^ due to C–O–C stretching vibrations, 1213 cm^−1^ due to C–C stretches; 1360 and 1452 cm^−1^ due to the bending and deformation vibration of –CH_3_ bonds, respectively; and 1754 cm^−1^ due to –C=O stretching vibrations [34]. The TP spectrum is characterized by a broad absorption band at 3000–3500 cm^−1^ that corresponds to O–H stretching vibrations [35,36]. The spectra of the PLA/TP composite nanofiber samples revealed that the introduction of the TPs resulted in noticeably stronger absorptions at 1087 cm^−1^ because of C–O-bond vibrations and at 1755 cm^−1^ because of C=O-bonds vibrations. Absorptions at 1613 cm^−1^, which are characteristic of the C=C bonds of phenyl groups, and absorptions at 821 cm^−1^, which are characteristic of mono-substituted phenyl moieties, were also observed [37]. These data indicate that the TPs reacted with PLA to alter the functional-group features of the PLA.

### 3.4. Mechanical Properties

The mechanical properties of the PLA/TP nanofibers were also examined; the results are displayed in Table 1, where it can be seen that the presence of the TPs resulted in a noticeable decrease in the tensile strength of each PLA/TP fiber compared to that composed of pure PLA. For instance, the tensile strength of the 2:1 sample (4.86 ± 2.3 MPa) was significantly lower than that of the PLA fiber (12.24 ± 4.5 MPa) (*p <* 0.05). The presence of the TPs also resulted in lower elongations at break for the PLA/TP fibers. For instance, the elongation at break of the 2:1 fiber (24.72% ± 4.34%) was significantly lower than that of the PLA fiber (57.28% ± 12.53%) (*p <* 0.05). This sharp decrease in tensile strength was ascribable to weakened PLA molecular bonding due to the presence of the TPs, while the significant decrease in elongation at break may have been explained by the embedment of TP molecules in the PLA polymer chains, which leads to a lower capacity of the PLA chains to stretch under drawing force and ultimately results in a lower elongation at break [31,38]. However, the tensile strength and elongation at break of the 3:1 fiber (9.28 ± 3.6 MPa and 50.36% ± 10.88%, respectively) were not significantly lower than those of the PLA. In other words, this fiber meets the mechanical requirements for food-packaging applications, which can be explained by the TP molecules acting as bridges between PLA molecular chains, thereby improving the tensile strength and the elongation at break of the composite nanofiber when compared to those of the 2:1 composite fiber. In a study of fibers formed from green tea extract, Siripatrawan et al. also demonstrated that the extracted TPs improved the tensile strength and elongation at break of the resulting composite fiber [39].

A decrease in solution viscosity leads to a higher degree of fiber stretching during the spinning process, resulting in finer fibers [33]. Therefore, when a small amount of TPs were added, the elongation at break and tensile strength of the fibers were reduced, but not obviously so. The viscosity of the solution continued to decrease with a gradually increasing concentration of TPs. Increasing the amount of TPs resulted in a decrease in fiber diameter; consequently, the tensile strength decreased significantly along with a deterioration in mechanical properties. At present, it seems that the mechanical properties of the fiber films may not meet the mechanical properties required for conventional packaging materials, and the mechanical properties of the modified fibers are inferior to those of the pure PLA fibers; however, the modified fibers were significantly improved in other ways. If the fibers are to be used for production itself, they will need to be subjected to a series of tests and post-processing; these will be reported following a subsequent study. Of course, the mechanical properties of the fiber membrane are sufficient for it to be applied to packaging that does not require high mechanical strengths and that can be selectively packaged. At present, there are many research reports on active fiber packaging materials, and many studies have yielded results that are not completely satisfactory; these need to be further explored and improved in subsequent experiments [20,40].

### 3.5. Hydrophilic–Hydrophobic Properties of PLA and PLA/TP Fibers

**Figure 4:**
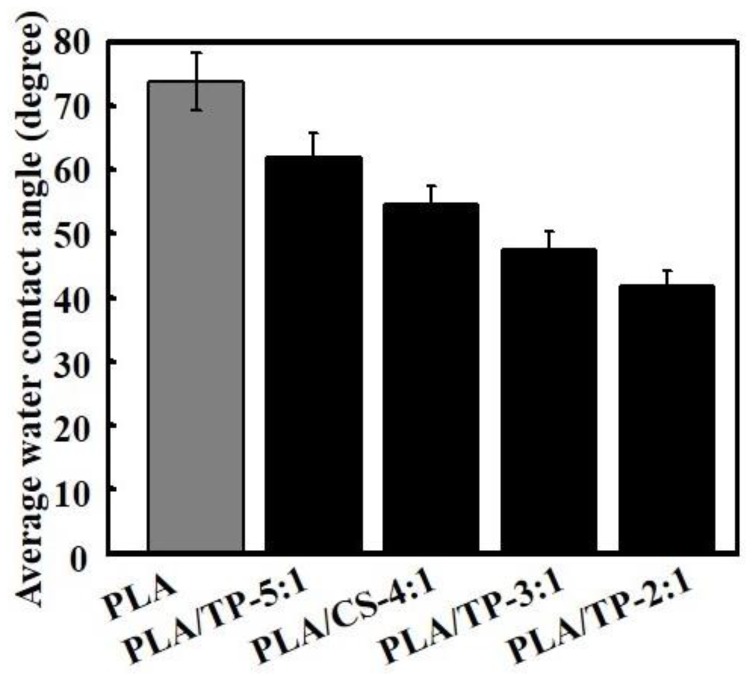
Contact angle of polylactic acid (PLA) fiber and PLA/tea polyphenol (TP) composite fibers.

Figure 4 shows the water contact angles on different PLA/TP fibers. The data indicate that PLA is hydrophobic; the water contact angle was 75.8° ± 8.4°. The PLA/TP fibers showed hydrophilicity, attributed to the incorporation of strongly hydrophilic TP, which significantly decreased the water contact angle of the composite nanofibers, particularly at high concentrations. The water contact angles on PLA/TP-5:1, PLA/TP-4:1, PLA/TP-3:1, and PLA/TP-2:1 fibers were 65.8° ± 7.5°, 54.6° ± 6.6°, 48.1° ± 5.7°, and 30.3° ± 4.1°, respectively. Biocompatible TP has strong sterilizing function, which is attributed to the α-phenylbenzopyran structure in TP [41].

### 3.6. Release of TP from PLA/TP Fibers into Different Food Simulants

**Figure 5:**
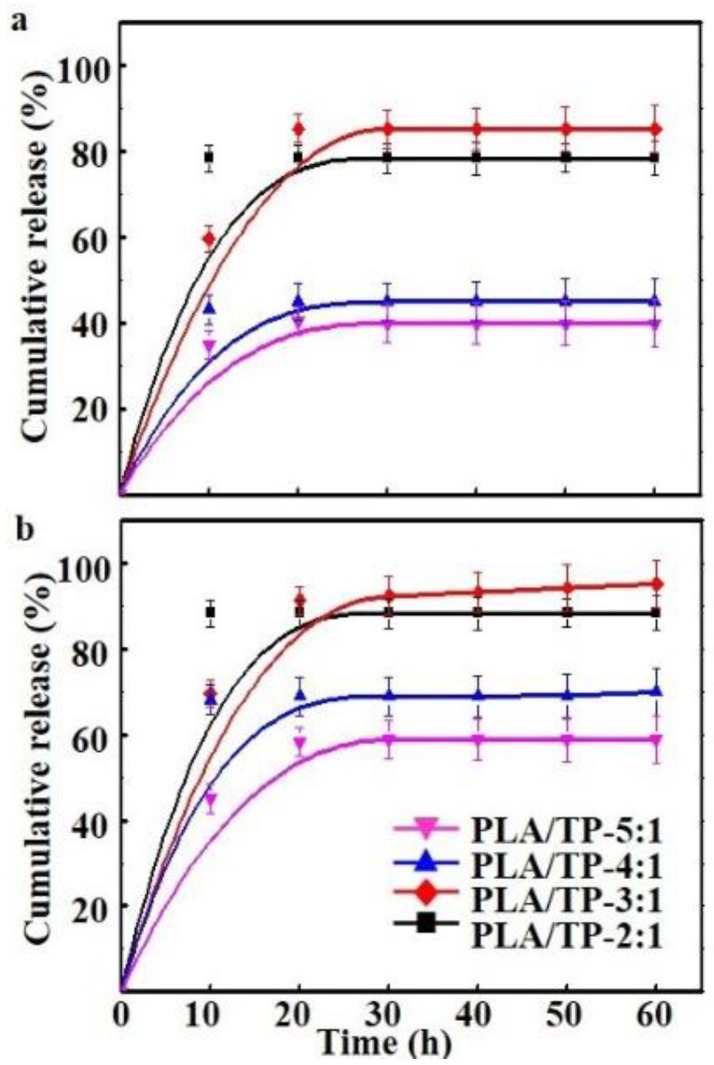
Release of tea polyphenols (TPs) from polylactic acid (PLA)/TP fibers in (**a**) 95% ethanol, and (**b**) 50% ethanol fatty food simulants as a function of time.

In both simulants, PLA/TP-5:1, PLA/TP-4:1, and PLA/TP-3:1 fibers had similar release profiles with an initial fast release followed by a sustained slow release (Figure 5a,b). TP released easily in different food simulants because of the excellent hydrophilicity of TP. As shown in Figure 5a, the TP release rate of the PLA/TP-5:1 and PLA/TP-4:1 fibers was significantly lower than for the PLA/TP-3:1 fiber; the cumulative TP release over 80% of the PLA/TP-3:1 fiber was the highest of all, which could be attributed to the increasing degradation rate of the matrix and the increasing content of TP staying on the surface of PLA, with more TP compared to the PLA/TP-5:1 and PLA/TP-4:1 fibers, according to studies of Liu et al. [30]. Furthermore, the PLA/TP-2:1 fiber had a more pronounced burst release in the initial 20 h than the others. This phenomenon could be explained by the fact that TP was mainly adsorbed on the surface of PLA and was distributed randomly in the fiber matrix by non-covalent interaction. Therefore, the burst release extent of the PLA/TP-2:1 fiber was the most acute.

In addition, the release of TP from the PLA/TP fibers in 50% ethanol was faster than that in the 95% ethanol fatty food simulants (Figure 5b). This difference might have been due to the higher swelling degree of the PLA/TP fibers in 50% ethanol, the diffusion of the simulant into film, and the relaxation of the film matrix [42].

### 3.7. DPPH Activity

**Figure 6:**
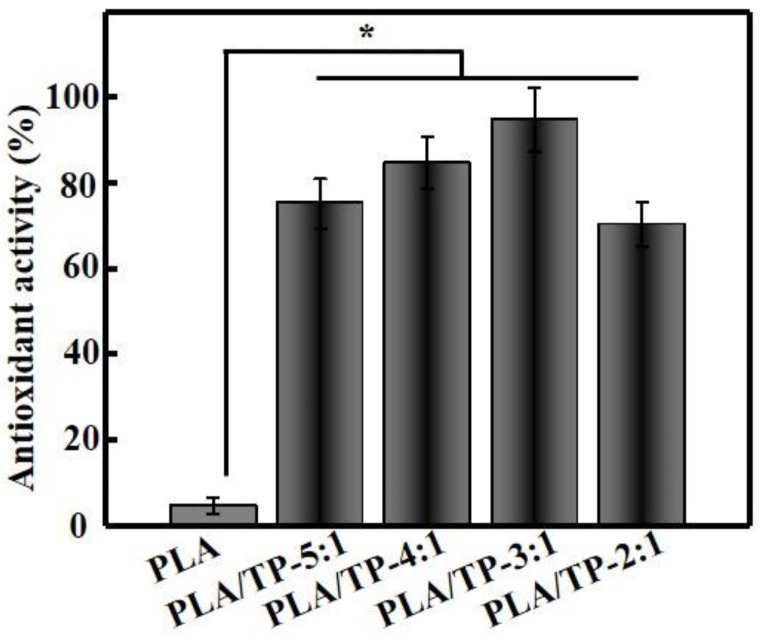
Antioxidant activities of various polylactic acid (PLA) and PLA/tea polyphenol (TP) composite fibers (* *p* < 0.05).

The DPPH radical-scavenging assay is now a standard technique for determining the antioxidant capacity of a substance. Figure 6 shows that the DPPH RSA of the PLA fiber (control) was very weak (4.58% ± 1.85%), while the PLA/TP-3:1 fibers exhibited pronounced antioxidant activity (95.07% ± 10.55%). As expected, the pure PLA did not show any antioxidant effect; however, it acted as a reservoir and protection system for the antioxidants investigated. The scavenging ability was related to the TP contents. Moreover, antioxidant activity was directly proportional to the TP content in the extract and encapsulates. PLA/TP-3:1 showed the highest antioxidant activity among the fibers, as the processing conditions of the encapsulation process affected the content and activity of TP. This increase also reflected the level of TPs released from the composite nanofiber; this level of release was closely related to the swelling of the composite nanofiber sample in the test reagent [42]. A similar result has also been reported by Parthasarathi et al. [43]. Figure 6 reveals that increases in TP content did not significantly affect the antioxidant capacity of the composite nanofiber, which may have been ascribable to the 3:1 composite nanofiber having a good overall morphology that was uniform in fiber diameter and to its high TP content that imparted a high DPPH radical-scavenging capacity. The morphology of the nanofiber worsened with further increases in TP content, with fiber diameters that were less uniform and that failed to contribute further to the swelling of the composite nanofiber and TP release. This explains why further increases in TP content did not lead to significant increases in DPPH radical-scavenging capacity.

### 3.8. Antibacterial Activity

**Figure 7:**
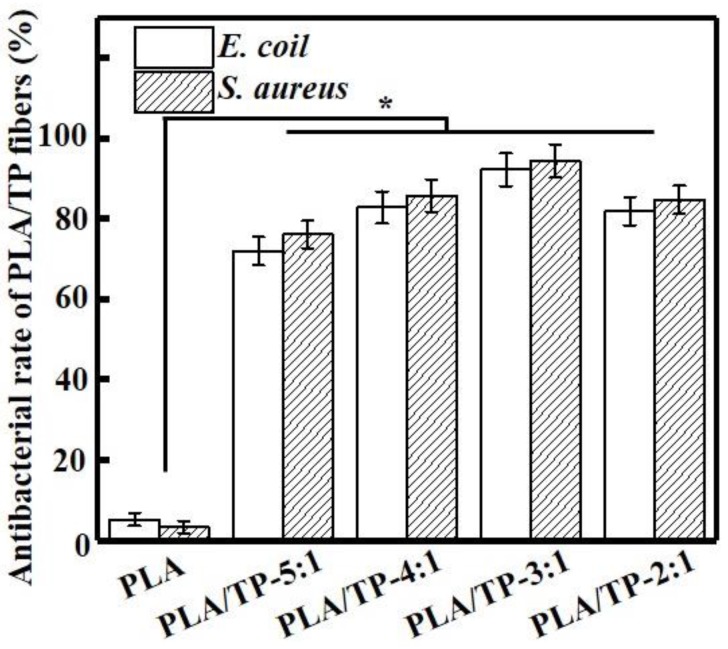
Antibacterial rates of different polylactic acid (PLA) and PLA/tea polyphenol (TP) composite fibers (* *p* < 0.05).

The improved vibration method was employed to determine the antimicrobial activities of the PLA/TP composite fibers against *E. coli* and *S. aureus* (Figure 7). All tested samples inhibited the growth of *E. coli* and *S. aureus*, while the PLA nanofiber did not display any notable antimicrobial activity (5.23% ± 1.85% and 3.11% ± 1.21%) because of the absence of an antimicrobial component. The antimicrobial activities against *E. coli* and *S. aureus* improved with increasing TP content. Li et al. also found that the presence of TPs noticeably improved the activities of dressing against *E. coli*; however, the antimicrobial mode of action of these TPs remains elusive [44]. Xiong et al. reported that TPs inhibit the growth of bacteria by increasing their endogenous oxidative stress reaction [45]. In our study, the PLA/TP-3:1 fiber demonstrated the best antimicrobial activity against both bacteria, with measured inhibitory activities of 92.26% ± 5.93% and 94.58% ± 6.53%, respectively (*p* < 0.05). It is interesting that the PLA/TP-3:1 fiber exhibited better antimicrobial activity against *S. aureus* than against *E. coli*, which was likely ascribable to the hydrophilic TP passing through the outer membrane through abundant pores in proteins that serve as hydrophilic transmembrane channels in *S. aureus*. In a study on the antibacterial activities of 10 plant polyphenols, Taguri et al. also demonstrated that TPs effectively inhibited *S. aureus* and had stronger antibacterial activities against gram-positive bacteria than gram-negative bacteria because of the presence of lipopolysaccharides in the latter species that decrease their sensitivities [46]. However, further studies need to be conducted to validate this hypothesis.

In addition, the antibacterial activities of the composite fiber membrane on *E. coli* and *S. aureus* were observed to increase with increasing amounts of TPs, but when PLA and TPs were added in a 2:1 ratio, the antibacterial activity decreased instead. There are three possible reasons for this phenomenon. Firstly, SEM revealed that the fibers exhibited broken fractures at a 2:1 PLA/TP addition ratio, while the fiber surfaces were smooth and the morphology was better at ratios other than 2:1. Secondly, at an addition ratio of 2:1, the collected fiber surfaces were no longer those of a smooth fiber membrane; consequently some of the TP particles did not recombine with the fiber and became dispersed on the surface of the fiber. Thirdly, TPs contain polyphenolic hydroxyl groups and are easily oxidized in air. They exhibit excellent antioxidant activities because they are preferentially oxidized prior to being protected [47]. Because the surface of a high-concentration TP fiber membrane has a certain amount of TPs that are not encapsulated by fibers, the antibacterial activity may also decline at a later time through exposure to the environment. Performance testing, in which this composite fiber is exposed to the environment for specific periods of time, will form part of subsequent studies. In summary, fiber morphology and TP distribution affect the antibacterial properties of these composite materials to a certain extent, which first leads to an increase in antibacterial activity, followed by a decrease. Our antioxidant testing studies revealed that the antioxidant activity of the composite fiber no longer increased with increasing amounts of added TPs after a certain value was reached.

## 4. Conclusions

PLA/TP composite nanofibers were successfully prepared by electrospinning. The fibers of the resulting composite materials were morphologically uniform, with fiber diameters that decreased with increasing TP content. The mechanical properties of the PLA/TP-3:1 nanofibers were slightly compromised compared to those of the PLA fibers, with a tensile strength and elongation at break of 9.28 ± 3.6 MPa and 50.36% ± 10.88%, respectively, which meet the requirements for food-packaging applications. The PLA/TP-3:1 composite fiber also exhibited the strongest DPPH RSA (95.07% ± 10.55%) and good antimicrobial activities against *E. coli* and *S. aureus* (92.26% ± 5.93% and 94.58% ± 6.53%, respectively). Therefore, PLA/TP composite nanofibers have the potential to be alternative bioactive materials for food-packaging applications.

## Figures and Tables

**Table 1:** Mechanical properties of different polylactic acid (PLA) and PLA/tea polyphenol (TP) composite fibers.

Sample	Tensile Strength	Breakage Elongation
PLA	12.24 ± 4.5a	57.28 ± 12.53a
PLA/TP-5:1	10.45 ± 4.2a	53.09 ± 11.75a
PLA/TP-4:1	9.87 ± 3.8a	51.71 ± 11.01a
PLA/TP-3:1	9.28 ± 3.6a	50.36 ± 10.88a
PLA/TP-2:1	4.86 ± 2.3b	24.72 ± 4.34b

Note: Different letters in the same column indicate significant differences (*p* < 0.05); the same letters in the same column represent non-significant differences (*p* > 0.05).
